# Tumor microbiome analysis provides prognostic value for patients with stage III colorectal cancer

**DOI:** 10.3389/fonc.2023.1212812

**Published:** 2023-10-26

**Authors:** Jae Hyun Kim, Jongwook Yu, Dong Keon Kim, Seunghun Lee, Seung Hyun Lee, Byung Kwon Ahn, Tae Il Kim, Seun Ja Park

**Affiliations:** ^1^ Department of Internal Medicine, Kosin University College of Medicine, Busan, Republic of Korea; ^2^ Department of Internal Medicine, Yonsei University College of Medicine, Seoul, Republic of Korea; ^3^ Department of Colorectal Surgery, Kosin University College of Medicine, Busan, Republic of Korea; ^4^ Brain Korea 21 Project for Medical Science, Yonsei University College of Medicine, Seoul, Republic of Korea

**Keywords:** colorectal cancer, microbiome, tissue, prognosis, tumor

## Abstract

**Introduction:**

Although patients with colorectal cancer (CRC) can receive optimal treatment, the risk of recurrence remains. This study aimed to evaluate whether the tumor microbiome can be a predictor of recurrence in patients with stage III CRC.

**Methods:**

Using 16S rRNA gene sequencing, we analyzed the microbiomes of tumor and adjacent tissues acquired during surgery in 65 patients with stage III CRC and evaluated the correlation of the tissue microbiome with CRC recurrence. Additionally, the tumor tissue microbiome data of 71 patients with stage III CRC from another center were used as a validation set.

**Results:**

The microbial diversity and abundance significantly differed between tumor and adjacent tissues. In particular, *Streptococcus* and *Gemella* were more abundant in tumor tissue samples than in adjacent tissue samples. The microbial diversity and abundance in tumor and adjacent tissues did not differ according to the presence of recurrence, except for one genus in the validation set. Logistic regression analysis revealed that a recurrence prediction model including tumor tissue microbiome data had a better prediction performance than clinical factors (area under the curve [AUC] 0.846 vs. 0.679, p = 0.009), regardless of sex (male patients: AUC 0.943 vs. 0.818, p = 0.043; female patients: AUC 0.885 vs. 0.590, p = 0.017). When this prediction model was applied to the validation set, it had a higher AUC value than clinical factors in female patients.

**Conclusion:**

Our results suggest that the tumor microbiome of patients with CRC be a potential predictor of postoperative disease recurrence.

## 1 Introduction

Colorectal cancer (CRC) is the second leading cause of cancer deaths worldwide. Compared with the 2020 estimates, the global burden of CRC is predicted to increase by 63% in 2040 (1). Moreover, the incidence of early-onset CRC (before age 50 years) is increasing in high-income countries (2). For resectable non-metastatic CRC, colectomy with en bloc removal of regional lymph nodes is the preferred treatment; however, several studies reported that approximately 25–30% of patients with stage III CRC experienced disease recurrence within the first 5 years after surgery (3–6). In addition, although adjuvant chemotherapy has demonstrated benefits in patients with stage III CRC, it can reduce the risk of recurrence by only approximately 30% (7, 8). The mortality rates for CRC are consistently higher in men compared to women across different regions worldwide, with men having a mortality rate approximately 25% higher than women (9). Several retrospective studies have shown that female CRC patients typically have longer survival rates than males (10–12). However, some studies have failed to find any survival benefit for women (13). Several prognostic factors for CRC recurrence have been recognized, including a poorly differentiated histology, greater tumor depth, higher number of positive lymph nodes, lymphovascular invasion, perineural invasion, and tumor budding (14–17). In contrast, high microsatellite instability (MSI) and abundant tumor-infiltrating T-cells have been associated with a favorable prognosis in patients with CRC (18–20). Recently, the detection of circulating tumor DNA after surgery has been suggested as a predictor of a high risk of recurrence (21, 22). Nevertheless, a more precise prediction of the risk of CRC recurrence after surgery is still required in clinical practice.

Emerging evidence has demonstrated the microbial composition and ecological changes in patients with CRC and the roles of several bacteria in colorectal carcinogenesis and treatment (23). The gut microbiome, which includes *Faecalibacterium*, *Akkermansia*, and *Bifidobacterium* species, is expected to play an important role in mediating the outcomes of chemotherapy and immunotherapy in patients with melanoma and lung cancer, as it affects immune system activation and tumor responses to treatment (24–26). In particular, the presence of abundant *Fusobacterium nucleatum* (*F. nucleatum*) DNA in tissues has been associated with worse clinical outcomes in patients with CRC (27). One study of patients with pancreatic cancer demonstrated that the diversity and composition of the tumor microbiome are important determinants of long-term survival (28). A recent study of patients with CRC showed that two pathogenic bacteria, *F. nucleatum* and *Bacteroides fragilis* (*B. fragilis*), were more abundant in patients without recurrence than in those with recurrence (29). However, the association between the tumor microbiome and clinical outcomes in patients with CRC remains unclear.

We designed this study to investigate the potential role of the tumor microbiome in predicting postoperative recurrence in patients with stage III CRC. To verify the results, we also analyzed the tumor microbiome data of patients with stage III CRC from another center.

## 2 Materials and methods

### 2.1 Patients and sample collection

Two pairs of tumor tissues and adjacent normal-appearing mucosal tissues (hereinafter “adjacent tissues”) from patients with CRC who underwent colorectal resection at Kosin University Gospel Hospital (Busan, Republic of Korea) were previously collected and stored immediately in a deep freezer (−80°C). From these samples, we selected and analyzed tumor and adjacent tissues from patients with stage III CRC who underwent adjuvant chemotherapy. Patients with pathological stage I or II CRC who had clinical stage III disease before surgery, those with < 3 months of adjuvant chemotherapy, and those with < 24 months of follow-up were excluded from the analysis. Further, tumor tissue samples from patients with stage III CRC who underwent surgery and adjuvant chemotherapy at Yonsei University Severance Hospital (Seoul, Republic of Korea) were used as a validation set. Detailed clinical data, such as age, sex, height, weight, ABO blood type, history of smoking and alcohol drinking, family history of CRC, comorbid diseases, tumor location, histology, lymphovascular invasion, perineural invasion, Kirsten rat sarcoma viral oncogene homolog (KRAS) mutation, MSI status, T stage, N stage, and laboratory findings (including carcinoembryonic antigen [CEA] level), were assessed. The study protocol was reviewed and approved by the institutional review board of Kosin University Gospel Hospital (approval no. KUGH 2021-01-028).

### 2.2 Adjuvant chemotherapy and definition of recurrence

Patients with CRC who underwent colorectal resection received were given either FOLFOX, CAPEOX, or FL as adjuvant chemotherapy for a duration of 6 months. The FOLFOX regimen includes intravenous administration of oxaliplatin 85 mg/m^2^, leucovorin 400 mg/m^2^, and a bolus of 5-fluorouracil 400 mg/m^2^ on day 1. This is followed by a continuous infusion of 5- fluorouracil 1200 mg/m^2^/day for 2 days. The treatment cycle is repeated every 2 weeks. The CAPEOX regimen includes intravenous administration of oxaliplatin 130 mg/m^2^ on day 1 and oral administration of capecitabine 1000 mg/m^2^ twice a day for 14 days. The treatment cycle is repeated every 3 weeks. The FL regimen consists of intravenous administration of leucovorin 400 mg/m^2^, and a bolus of 5-fluorouracil 400 mg/m^2^ on day 1. This is followed by a continuous infusion of 5- fluorouracil 1200 mg/m^2^/day for 2 days.

The recurrence of CRC was diagnosed on endoscopic biopsy, surgical resection, and/or radiological imaging study. In this study, we defined recurrence as both locoregional and distant recurrence. Locoregional recurrence was defined as a recurrence at the site of original surgical resection or at the draining lymph nodes. Distant recurrence was defined as a recurrence of CRC developing spread to distant sites including the liver, lung, peritoneum, ovaries, adrenal glands, bone, and brain.

### 2.3 DNA extraction and bacterial 16S rRNA sequencing

The samples collected at Kosin University Gospel Hospital were transported to Hecto Healthcare Co., Ltd. (Seoul, Korea) and immediately frozen at −80°C. Microbial DNA was extracted using the Maxwell^®^ RSC PureFood GMO and Authentication Kit (Promega, Madison, WI, USA) according to the manufacturer’s instructions. To determine DNA concentrations, we used an ultraviolet–visible spectrophotometer (NanoDrop 2000c; Thermo Fisher Scientific, Waltham, MA, USA). QuantiFluor^®^ ONE dsDNA System (Promega) was used for quantification. The DNA samples were stored at −20°C until required for experiments. A sequencing library was prepared according to the Illumina 16S Metagenomic Sequencing Library Preparation Guide (Illumina, San Diego, CA, USA). The V3–V4 region of the bacterial 16S rRNA gene was amplified using primer sets F319 (5′-TCGTCGGCAGCGT-CAGATGTGTATAAGAGACAGCCTACGG-GNGGCWGCAG-3′) and R806 (5′-GTCTCGTGGGCTCGGAGATGTGTATAAGAGAC-AGGACTACHVGGGTATC-TAATCC-3′). The amplified products were purified using Agencourt^®^ AMPure XP beads (Beckman Coulter, Brea, CA, USA), and the quality of the library was confirmed using the Bioanalyzer 2100 system (Agilent, Santa Clara, CA, USA). The pooled libraries were sequenced with 300-bp paired-end reads on the MiSeq platform using the MiSeq version 3 Reagent Kit (Illumina). To prevent contamination, all experimental procedures were conducted inside a biosafety cabinet (BSC). DNA extraction was performed using sterile disposable Petri dishes and surgical blades to cut the sample into appropriate sizes while it was still frozen on dry ice. During the analysis stage, library pooling was performed by mixing Phix control at a 30% ratio with filtered real sequences used as raw data. The resulting data was then subjected to quality filtering, denoising, and sequencing error removal using QIIME2 software before proceeding with further analysis.

### 2.4 Data analysis and statistical analysis

Raw sequencing data were processed using the Quantitative Insight into Microbial Ecology software package 2 (QIIME 2, version 2021.4; http://qiime2.org). Denoising was performed using the Deblur algorithm, and a taxonomy table was created using the SILVA database (version 138). The non-archaeal/bacterial sequences were removed according to the taxonomic classification results. FASTQ reads were filtered, trimmed, and merged in DADA2 to generate a table of amplicon sequence variants. Taxonomy was assigned to the amplicon sequence variants using a naive Bayes classifier and compared to the SILVA version 138.99 reference database. Alpha diversity was assessed using the Shannon index, Chao1 index, Simpson index, and observed operational taxonomic units, whereas beta diversity was evaluated using principal coordinate analysis based on the Bray–Curtis distance. These analyses were performed using QIIME 2 and R (version 4.1.3; R Foundation for Statistical Computing, Vienna, Austria). To compare the taxa, we selected only those with a mean relative abundance greater than or equal to 1%. Data visualization was performed using the ggplot2 package in R, and statistical analysis was conducted using the Wilcoxon signed rank test and PERMANOVA from the vegan package. Linear discriminant effect size analysis was performed using the online platform, Galaxy (https://huttenhower.sph.harvard.edu/galaxy).

The patients’ demographic and clinical data were compared using Student’s t-test and Fisher’s exact test. Continuous data with a normal distribution are expressed as mean ± standard deviation, and categorical data are presented as numbers (percentage). The Wilcoxon signed-rank test was used to compare microbial abundance between tumor and adjacent tissues, as well as according to the presence of recurrence. Logistic regression analysis was performed to evaluate factors predicting disease recurrence. The ‘glm’ function in R was used to fit a logistic regression model to our data, including predictors such as clinical variables and microbiome to predict the binary outcome variable of recurrence. The ‘step’ function was then used to perform backward selection and select the final model. Receiver operating characteristic (ROC) and area under the curve (AUC) analyses were performed to estimate the thresholds of variables. A random forest model was used to assess the mean decrease in the Gini coefficient. To control for the false discovery rate (FDR), statistical significance was determined using the Benjamini-Hochberg procedure with a threshold of FDR-adjusted p value < 0.05. All statistical analyses were performed using R.

## 3 Results

### 3.1 Baseline characteristics and evaluation of clinical variables affecting recurrence

Patients with stage III CRC who underwent surgery followed by adjuvant chemotherapy at Kosin University Gospel Hospital (65 patients, discovery set) and Yonsei University Severance Hospital (71 patients, validation set) were enrolled in this study. The baseline characteristics of the patients are summarized in **Table 1**. The mean age in the discovery set was younger than that in the validation set (60.0 ± 9.3 vs. 64.7 ± 11.4 years, *p* = 0.010). Additionally, the discovery set had a higher prevalence of current smokers (27.7% vs. 9.9%, *p* = 0.027) and lymphovascular invasion (63.1% vs. 36.6%, *p* = 0.004) compared to the validation set. In the discovery set, 60 patients (92.3%) received FOLFOX and 5 patients (7.7%) received CAPEOX. In the validation set, 59 patients (83.1%) received FOLFOX, 8 patients (11.3%) received CAPEOX, and 4 patients (5.6%) received FL. All of the patients received treatment for a minimum of 5 months or more.

**Table 1:** Baseline characteristics.

Characteristics	Discovery set (n = 65)	Validation set (n = 71)	*p* Value
Age (years)	60.0 ± 9.3	64.7 ± 11.4	0.010
Sex			0.335
Male	34 (52.3)	44 (62.0)	
Female	31 (47.7)	27 (38.0)	
Height (cm)	161.6 ± 9.2	163.1 ± 8.8	0.325
Weight (kg)	61.2 ± 11.5	63.2 ± 11.3	0.303
BMI (kg/m^2^)	23.3 ± 3.4	23.7 ± 3.5	0.552
ABO blood type			0.096
A	34 (52.3)	30 (42.3)	
B	9 (13.8)	21 (29.6)	
O	14 (21.5)	16 (22.5)	
AB	8 (12.3)	4 (5.6)	
Smoking			0.027
None	29 (44.6)	41 (57.7)	
Past	18 (27.7)	23 (32.4)	
Current	18 (27.7)	7 (9.9)	
Alcohol drinking			0.299
None	26 (40.0)	33 (46.5)	
Past	23 (35.4)	28 (39.4)	
Current	16 (24.6)	10 (14.1)	
Family history	4 (6.2)	7 (9.9)	0.633
Comorbid diseases			0.204
None	36 (55.4)	34 (47.9)	
DM	11 (18.5)	17 (23.9)	
HTN	17 (33.8)	34 (47.9)	
Dyslipidemia	1 (1.5)	3 (4.2)	
Vascular disorders	7 (10.5)	4 (5.6)	
Hepatitis C	1 (1.5)	0 (0)	
Stomach cancer	1 (1.5)	1 (1.4)	
Tumor location			0.197
Right colon	23 (35.4)	36 (50.7)	
Left colon	28 (43.1)	23 (32.4)	
Rectum	14 (21.5)	12 (16.9)	
Histology			0.794
Well differentiated	4 (6.2)	7 (9.9)	
Moderately differentiated	54 (83.1)	58 (81.7)	
Poorly differentiated	5 (7.7)	5 (7.0)	
SRC/mucinous	2 (3.1)	1 (1.4)	
Lymphovascular invasion	41 (63.1)	26 (36.6)	0.004
Perineural invasion	15 (23.1)	14 (19.7)	0.789
KRAS mutation	11 (42.3)	25 (39.1)	0.962
MSI status			0.223
MSS	40 (61.5)	61 (85.9)	
MSI-low	1 (1.5)	0 (0.0)	
MSI-high	5 (7.8)	3 (4.2)	
N/A	19 (29.2)	7 (9.9)	
Tumor stage			0.060
IIIA	4 (6.2)	10 (14.1)	
IIIB	45 (69.2)	53 (74.6)	
IIIC	16 (24.6)	8 (11.3)	
Adjuvant chemotherapy regimen			0.145
FOLFOX	60 (92.3)	59 (83.1)	
CAPEOX	5 (7.7)	8 (11.3)	
FL	0 (0.0)	4 (5.6)	
CEA (ng/mL)	12.0 ± 18.7	8.2 ± 17.1	0.218

Values are presented as n (%) or mean ± standard deviation.

BMI, body mass index; DM, diabetes mellitus; HTN, hypertension; SRC, signet ring cell carcinoma; KRAS, Kirsten rat sarcoma viral oncogene homolog; MSI, microsatellite instability; MSS, microsatellite stable; N/A, non-available; FOLFOX, consists of oxaliplatin, leucovorin, and 5-fluorouracil; CAPEOX, consists of oxaliplatin and capecitabine; FL, consists of 5-fluorouracil and leucovorin; CEA, carcinoembryonic antigen.

**Figure 1:**
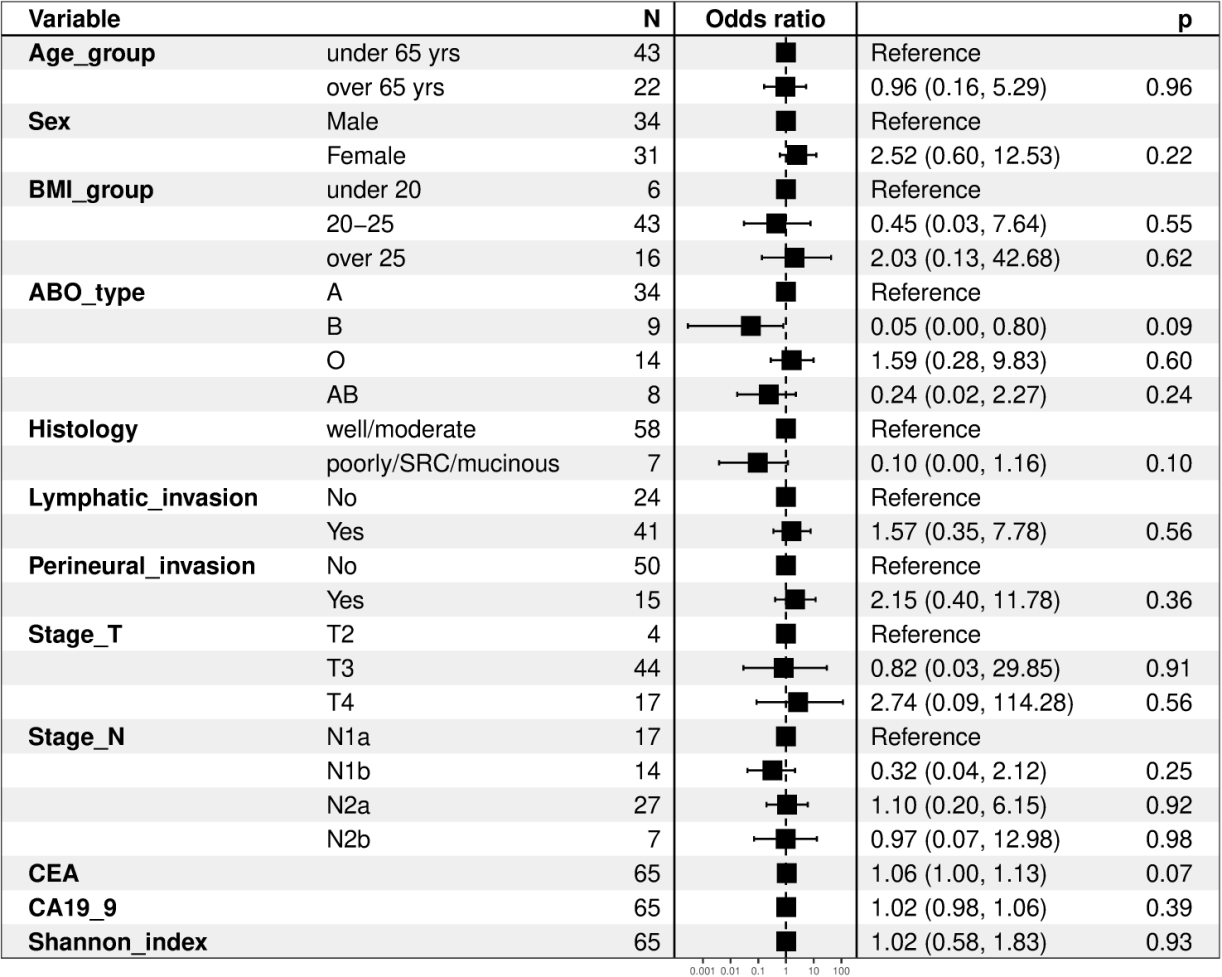
Forest plots of clinical factors as predictors of tumor recurrence. BMI, body mass index; SRC, signet ring cell carcinoma; CEA, carcinoembryonic antigen; CA19-9, carbohydrate antigen 19-9.

We compared the clinical variables according to the presence of recurrence, and no differences were observed in all factors, including tumor location, histology, lymphovascular invasion, perineural invasion, KRAS mutation, MSI status, T stage, N stage, and laboratory findings (**Table 2**). We evaluated clinical factors as predictors of tumor recurrence; however, none of the factors were found to be significant (**Figure 1**).

**Table 2:** Comparison between patients with and without recurrence.

	Discovery set			Validation set		
Characteristics	No recurrence (n = 40)	Recurrence (n = 25)	*p* Value	No recurrence (n = 52)	Recurrence (n = 19)	*p* Value
Tumor location			0.828			0.640
Rectum	13 (32.5)	10 (40.0)		25 (48.1)	11 (57.9)	
Left colon	18 (45.0)	10 (40.0)		17 (32.7)	6 (31.6)	
Right colon	9 (22.5)	5 (20.0)		10 (19.2)	2 (10.5)	
Histology			0.739			0.288
Well differentiated	3 (7.5)	1 (4.0)		6 (11.5)	1 (5.3)	
Moderately differentiated	32 (80.0)	22 (88.0)		43 (82.7)	15 (78.9)	
Poorly differentiated	4 (10.0)	1 (4.0)		3 (5.8)	2 (10.5)	
SRC/mucinous	1 (2.5)	1 (4.0)		0 (0.0)	1 (5.3)	
Lymphovascular invasion	24 (60.0)	17 (68.0)	0.699	19 (36.5)	7 (36.8)	1.0
Perineural invasion	7 (17.5)	8 (32.0)	0.295	8 (15.4)	6 (31.6)	0.237
KRAS mutation	8 (20.0)	3 (12.0)	0.354	17 (32.7)	8 (42.1)	0.618
MSI status			0.095			0.401
MSS	21 (52.5)	19 (100)		45 (86.5)	16 (84.2)	
MSI-low	1 (2.5)	0 (0)		3 (5.8)	0 (0.0)	
MSI-high	5 (12.5)	0 (0)		4 (7.7)	3 (15.8)	
N/A	13 (32.5)	6 (24.0)				
T stage			0.967			0.719
T1/2	3 (7.5)	1 (4.0)		8 (15.4)	2 (10.5)	
T3/4	37 (92.5)	24 (96.0)		44 (84.6)	17 (89.5)	
N stage			0.829			0.206
N1a/b	20 (50.0)	11 (44.0)		43 (82.7)	13 (68.4)	
N2a/b	20 (50.0)	14 (56.0)		9 (17.3)	6 (31.6)	
Hemoglobin (g/dL)	12.6 ± 1.8	12.7 ± 2.2	0.901			
White blood cells (×10^3^/µL)	7.3 ± 1.9	6.8 ± 2.0	0.341			
Platelets (×10^3^/µL)	265.7 ± 92.5	255.2 ± 74.4	0.637			
Glucose (mg/dL)	116.5 ± 59.6	118.4 ± 43.5	0.894			
HbA1c (%)	7.4 ± 2.4	7.1 ± 2.0	0.795			
Albumin (g/dL)	4.1 ± 0.4	4.1 ± 0.4	0.768			
HS-CRP (mg/dL)	0.8 ± 2.0	2.0 ± 4.2	0.234			
Cholesterol (mg/dL)	174.1 ± 29.5	170.7 ± 35.2	0.707			
HDL (mg/dL)	46.4 ± 11.5	46.1 ± 12.4	0.918			
Triglyceride (mg/dL)	92.6 ± 36.9	103.1 ± 38.8	0.336			
LDL (mg/dL)	108.4 ± 26.5	105.0 ± 31.4	0.678			
LDH (IU/L)	338.5 ± 63.6	362.5 ± 87.8	0.210			
CEA (ng/mL)	8.8 ± 12.0	17.1 ± 25.5	0.142	7.3 ± 16.3	10.6 ± 19.3	0.484
CA 19-9 (U/mL)	13.1 ± 26.9	18.6 ± 18.5	0.375			

Values are presented as n (%) or mean ± standard deviation.

SRC, signet ring cell carcinoma; KRAS, Kirsten rat sarcoma viral oncogene homolog; MSI, microsatellite instability; MSS, microsatellite stable; N/A, non-available; HbA1c, hemoglobin A1c; HS-CRP, high-sensitivity C-reactive protein; CEA, carcinoembryonic antigen; CA 19-9, carbohydrate antigen 19-9; HDL, high-density lipoprotein; LDL, low-density lipoprotein ; LDH, lactate dehydrogenase.

### 3.2 Microbiome differences between adjacent and tumor tissues

**Figure 2:**
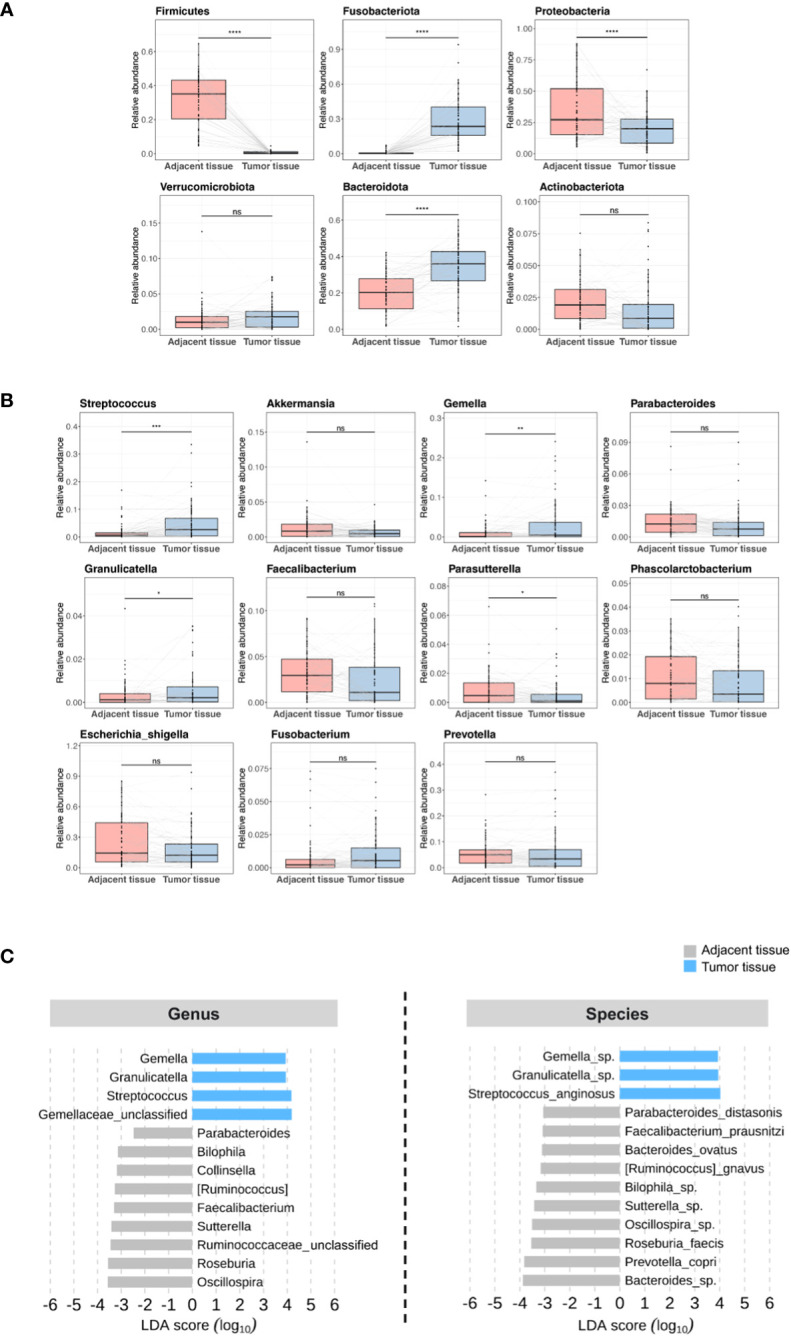
Microbial abundance between adjacent and tumor tissues in patients in the discovery set. **(A)** Phylum level. **(B)** Genus level. **(C)** Linear discriminant analysis effect size. ns, non-significant; LDA, linear discriminant analysis. '*', p < 0.05; '**', p < 0.01; '***', p < 0.001; '****', p < 0.0001.

On the basis of previous results (27, 28), we hypothesized that the tissue microbiome of patients with CRC could be a predictor of tumor recurrence after surgery. We focused on the individual differences in the microbiome and attempted to evaluate the possibility that the tissue microbiome can predict recurrence in patients with stage III CRC who underwent surgery and adjuvant chemotherapy. We compared the microbiome differences between adjacent and tumor tissues in patients in the discovery set. Alpha diversity was not different but beta diversity was significantly different between the two tissues, and the taxonomic composition showed differences at the phylum, genus, and species levels (**Figure S1**). Microbial abundance was remarkably different between adjacent and tumor tissues. At the phylum level, Fusobacteriota, Verrucomicrobiota, and Bacteroidota were more abundant in tumor tissue samples (**Figure 2A**). At the genus level, *Streptococcus* and *Gemella* were more abundant in tumor tissue samples (**Figure 2B**). In contrast, the phyla Firmicutes, Proteobacteria, and Actinobacteriota (**Figure 2A**), and the genera *Parabacteroides*, *Faecalibacterium*, and *Parasutterella* were more abundant in adjacent tissue samples (**Figure 2B**). Further, linear discriminant effect size analysis confirmed that the microbial abundance in adjacent tissues was distinct from that in tumor tissues (**Figure 2C**).

### 3.3 Microbiome differences according to the presence of recurrence

**Figure 3:**
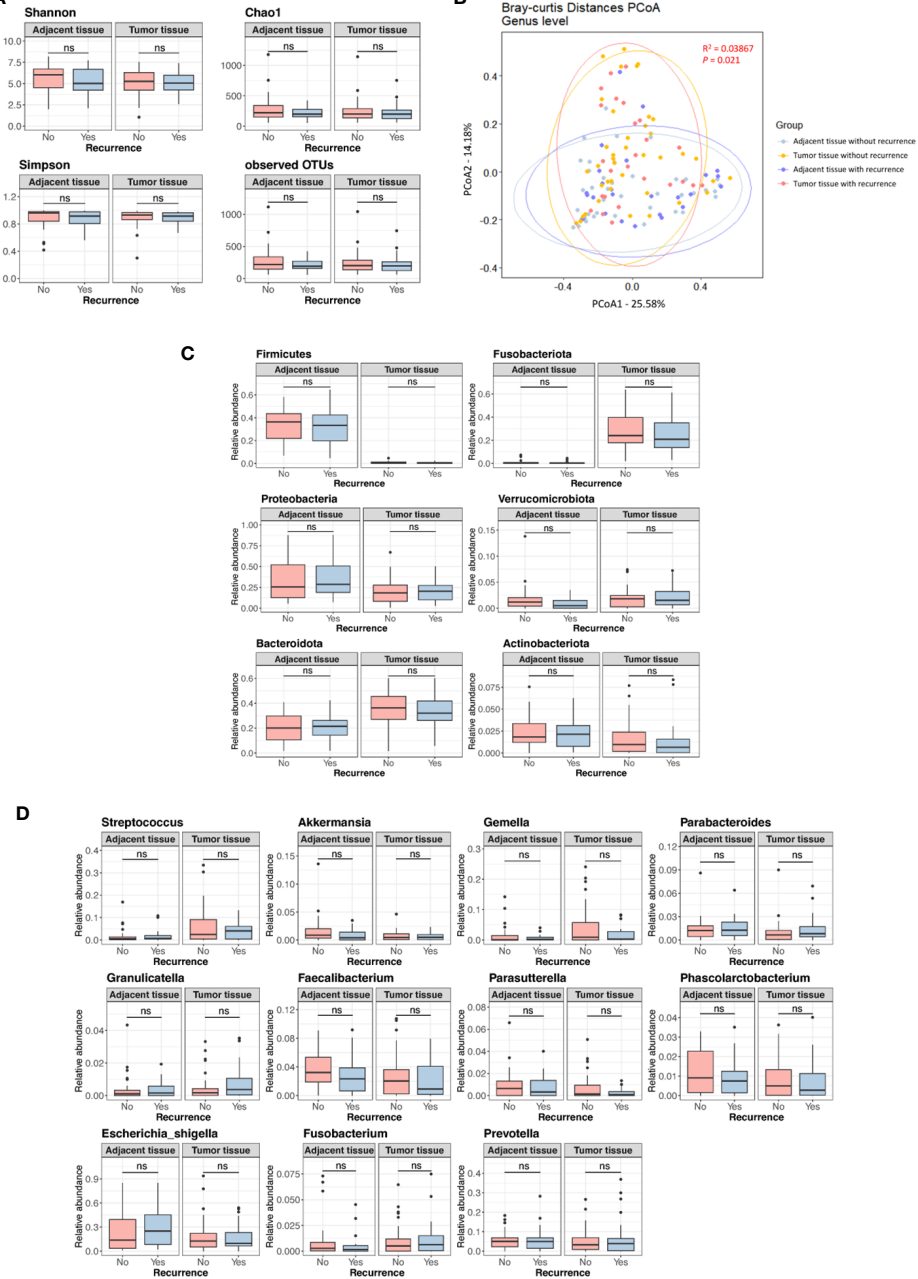
Microbial diversity and abundance in adjacent and tumor tissues according to the presence of recurrence in the discovery set. **(A)** Alpha diversity. **(B)** Beta diversity. **(C)** Phylum level. **(D)** Genus level. OTUs, operational taxonomy units; PCoA, principal coordinate analysis; ns, non-significant.

**Figure 4:**
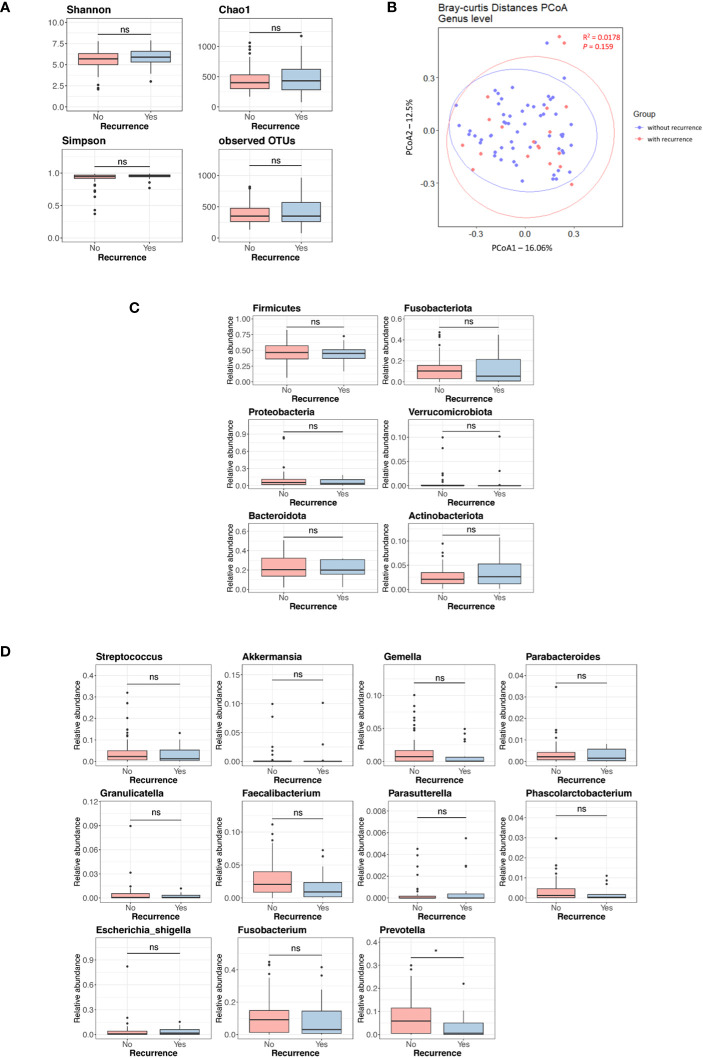
Microbial diversity and abundance in tumor tissues according to the presence of recurrence in the validation set. **(A)** Alpha diversity. **(B)** Beta diversity. **(C)** Phylum level. **(D)** Genus level. OTUs, operational taxonomy units; PCoA, principal coordinate analysis; ns, non-significant. '*', p < 0.05.

**Figure S2** displays the Kaplan-Meier curves for overall survival and disease-free survival differences between the discovery set and validation set. We assessed differences in the tissue microbiome according to the presence of recurrence. As shown in **Figure S3**, the taxonomic composition of the tissue microbiome was not different at the phylum, genus, and species levels between patients with and without recurrence in both the discovery and validation sets. In the discovery set, alpha diversity, beta diversity, and microbial abundance at the phylum and genus levels in adjacent and tumor tissues were not significantly different according to the presence of recurrence (**Figure 3**). Similar results were obtained when the data were divided into male and female groups (**Figure S4**). In the validation set, alpha and beta diversities did not differ according to the presence of recurrence, and microbial abundance at the phylum and genus levels were also not different, except for the genus *Prevotella* (**Figure 4**). Similar results were obtained when the data were divided into male and female groups; however, *Prevotella* was more abundant in tumor tissue samples from male patients without recurrence (**Figure S5**).

### 3.4 Generation and validation of a prediction model for CRC recurrence

Although we found no significant differences in tissue microbial diversity and abundance between patients with and without recurrence, we attempted to generate a recurrence prediction model including microbiome data using logistic regression analysis in the discovery set. When the analysis was performed by combining clinical factors (age, CEA level, histology, lymphovascular invasion, perineural invasion, stage T, and stage N) and tumor microbiome data (selecting only the genera with a relative abundance greater than or equal to 1%), we found that CEA level, T stage, and perineural invasion (among clinical factors), as well as the tumor tissue microbiome (including *Gemella*, *Parabacteroides*, *Parasutterella*, and *Prevotella*) were significant. We obtained the following estimation formula for the prediction model (see **Supplementary Data**):


f(x)=− 4.29796+0.04667*CEA level+1.08028∗T stage+1.47743∗perineural invasion−33.38073∗Gemella+28.07568∗Parabacteroides−141.75533*Parasutterella+7.85802∗Prevotella(Akaike information criteria:74.9, Nagelkerke R2: 47.2%)

**Figure 5:**
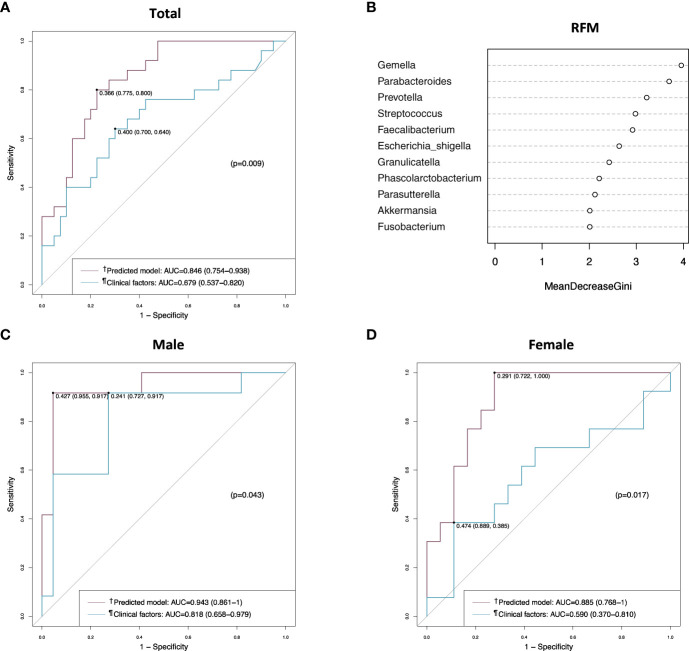
Receiver operating characteristic (ROC) curve and random forest model analyses in the discovery set. **(A)** ROC curves of the prediction model and the clinical factors in the total patients. **(B)** Random forest model evaluating tissue microbiomes. **(C)** ROC curves of the prediction model and the clinical factors in male patients. **(D)** ROC curves of the prediction model and the clinical factors in female patients. ^†^Includes clinical factors (CEA level, T stage, and perineural invasion) and tumor tissue microbiome (*Gemella*, *Parabacteroides*, *Parasutterella*, and *Prevotella*). ^¶^Includes CEA level, T stage, and perineural invasion. AUC, area under the curve; RFM, random forest model.

We applied the prediction model in generating the ROC curve and compared it to clinical factors (combination of CEA level, T stage, and perineural invasion) without microbiome. The AUC value of this model was 0.846 (95% confidence interval [CI], 0.754–0.938) in the total patients, and a good AUC value was obtained in both male and female patients (**Figure 5**). When compared with the ROC curve of clinical factors without microbiome, the prediction model showed a significantly better AUC value than clinical factors in the total patients (0.846 vs. 0.679, *p* = 0.009) (**Figure 5A**), regardless of sex (0.943-0.818, *p* = 0.043 in male; 0.885 vs. 0.590, *p* = 0.017 in female) (**Figures 5C, D**). In the random forest model analysis, *Gemella*, *Parabacteroides, and Prevotella* had a mean decrease in the Gini coefficient of > 3.0 (**Figure 5B**).

**Figure 6:**
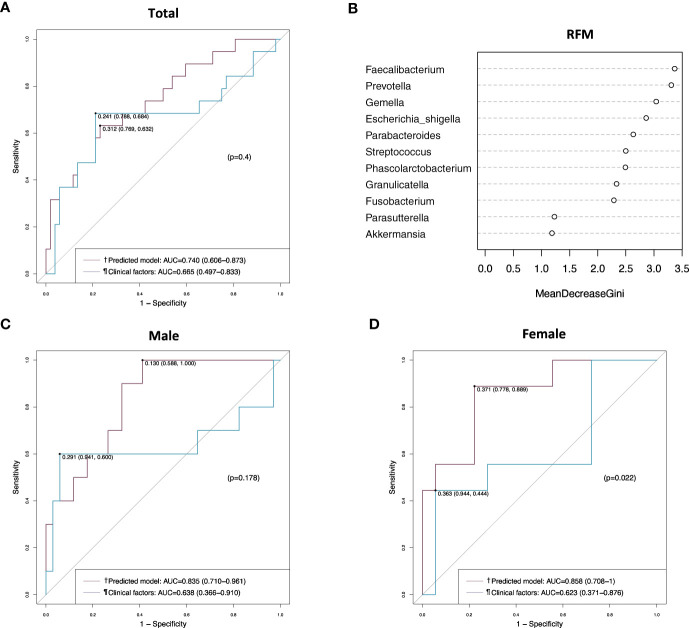
Receiver operating characteristic (ROC) curve and random forest model analyses in the validation set. **(A)** ROC curves of the prediction model and the clinical factors in the total patients. **(B)** Random forest model evaluating tissue microbiomes. **(C)** ROC curves of the prediction model and the clinical factors in male patients. **(D)** ROC curves of the prediction model and the clinical factors in female patients. ^†^Includes clinical factors (CEA level, T stage, and perineural invasion) and tumor tissue microbiome (*Gemella*, *Parabacteroides*, *Parasutterella*, and *Prevotella*). ^¶^Includes CEA level, T stage, and perineural invasion. AUC, area under the curve; RFM, random forest model.

When the prediction model was applied to the validation set, it showed an AUC value of 0.740 (95% CI, 0.606–0.873), which was not better than the AUC value of clinical factors without microbiome in the analysis of the total patients (**Figure 6A**). However, the prediction model showed a better AUC value than clinical factors in female patients (0.858 vs. 0.624, *p* = 0.022) (**Figure 6D**), but not in male patients (**Figure 6C**). In the random forest model analysis of the validation set, *Faecalibacterium*, *Prevotella* and *Gemella* had a mean decrease in the Gini coefficient of > 3.0 (**Figure 6B**).

## 4 Discussion

In the present study, we assessed a model combining clinical factors and tumor tissue microbiome data for predicting recurrence in patients with stage III CRC. This model showed better AUC values than clinical factors. Our data suggest that analysis of the tumor tissue microbiome combined with clinical factors may help predict recurrence in patients with CRC.

Recent studies have identified *Fusobacterium*, *Bacteroides*, *Peptostreptococcus*, *Gemella*, and *Parvimonas* as genera that are potentially associated with CRC, and emerging evidence has demonstrated their oncogenic functions; however, inter-individual variations in tumor-associated mucosal microbiome remain a barrier to elucidating the role of the microbiome in colorectal tumorigenesis. Concerning intra-individual variations in microbial patterns, several studies have shown that the microbiome structure of cancerous tissues significantly differs from that of the intestinal lumen, and that the microbiome of CRC tissues remarkably differs from that of adjacent tissues (30–32). Consistent with previous studies, our study showed significant differences in the beta diversity and abundance of microbiome between tumor and adjacent tissues. In particular, *Streptococcus* and *Gemella* were more abundant in tumor tissue samples than in adjacent tissue samples. An analysis of paired samples of CRC-adjacent mucosa and colonic mucosa from healthy controls showed differences in microbial community configurations (33). These results suggest that the microbial communities in the colorectal mucosa show distinct alterations according to the stage of colorectal carcinogenesis.

The observed association between the gut microbiome and clinical outcomes has raised the possibility that bacteria can serve as prognostic markers. Several studies reported that increased abundance of *F. nucleatum* and *B. fragilis* was associated with poor clinical outcomes and late-stage CRC (34, 35). In a recent study investigating the profiles of the gut mucosal microbiome in patients with CRC recurrence, a total of 17 bacteria were suggested as potential biomarkers for CRC recurrence and patient prognosis (36). In addition, the persistence of *F. nucleatum* after neoadjuvant chemoradiotherapy in patients with locally advanced rectal cancer was found to be correlated with high relapse rates (37). In the present study, we assessed microbial differences according to the presence of recurrence, and found no significant differences in microbial diversity and the abundance of each microbial group between patients with and without recurrence, except for one genus in the validation set. This lack of difference may be explained by the possibility that a network of numerous microbiomes, rather than the presence of a characteristic microbiome in tumor tissues, contributes to the development of recurrence.

We generated a prediction model for CRC recurrence by combining clinical factors and tumor tissue microbiome data. The model finally included several genera, such as *Gemella*, *Parabacteroides*, *Parasutterella*, and *Prevotella*. This prediction model had a good AUC value in patients with CRC regardless of sex and showed significantly better performance in predicting recurrence than the clinical factors. These results suggest that gut microbiome assessment has a potential role in predicting CRC recurrence; however, further studies with larger sample sizes are needed.

Adjuvant chemotherapy has demonstrated benefits in patients with stage III CRC, it can reduce the risk of recurrence by approximately 30% (7, 8). According to the NCCN guidelines, for low-risk (T1-3, N1) stage III CRC patients, CAPEOX (3 months) or FOLFOX (3-6 months), as well as other options like capecitabine (6 months) or 5-FU (6 months), are recommended. On the other hand, for high-risk (T4, N1-2; any T, N2) stage III CRC patients, the recommended options include CAPEOX (3-6 months) or FOLFOX (6 months), as well as other options like capecitabine (6 months) or 5-FU (6 months) (38). Liquid biopsy is a promising alternative strategy for directly evaluating circulating tumor DNA (ctDNA) from the blood. It aims to detect evidence of minimal residual disease, which could potentially be the source of a later clinical recurrence. Recently, in a study of 455 stage II CRC patients, ctDNA-guided management led to a reduced rate of adjuvant chemotherapy usage, and ctDNA-positive patients who received adjuvant chemotherapy exhibited a three-year recurrence-free survival of 86.4% (39). Although further research is needed, the combined analysis of liquid biopsy and tumor microbiome has the potential to offer more promising insights into predicting patient prognosis and determining the need for additional chemotherapy after surgery in stage III CRC patients.

The strength of our study is that the results obtained by analyzing tumor and adjacent tissue samples from one center were validated by comparing them with tumor tissue data from another center. However, our study had several limitations. First, the tumor tissue samples from the two centers were collected at different times and stored in different locations, which may have introduced heterogeneity in the results. Second, we could not compare the microbiomes of adjacent tissues in the validation set because no adjacent tissue data were collected from the other center. Third, the prediction model generated using the discovery set did not show a better AUC value than the clinical factors for the total patients and male patients in the validation set. We believe that this was due to data heterogeneity and the small number of samples. Fourth, the study’s sample size was small, which could reduce the reliability of our results. To overcome these limitations, further well-designed studies with larger sample sizes are needed.

In summary, we conducted a comprehensive investigation of the differences in microbial diversity and abundance between tumor and adjacent tissues, as well as their association with recurrence in CRC patients. Additionally, we developed a prediction model using tissue microbiome data to forecast postoperative recurrence. While the predictive performance of our model, measured by AUC values, did not surpass that of the clinical factors alone in the validation set, we did observe a relatively higher AUC value for the new model using microbiome data in female patients. However, we acknowledge the need for further research to explore potential gender-based differences in the microbiome profile’s predictive capacity for CRC recurrence. Therefore, the approach for the generalization of these findings should proceed with caution, and we refrain from unequivocally concluding that the tumor microbiome can predict postoperative disease recurrence in all patients. Nevertheless, we believe that our study contributes to emphasizing the importance of the tissue microbiome in diagnosing and predicting the recurrence of CRC.

## Data availability statement

The raw data supporting the conclusions of this article will be made available by the authors, without undue reservation.

## Ethics statement

The studies involving humans were approved by Kosin University Gospel Hospital (approval no. KUGH 2021-01-028). The studies were conducted in accordance with the local legislation and institutional requirements. The participants provided their written informed consent to participate in this study.

## Author contributions

JK: conceptualization, formal analysis, writing – original, and funding acquisition. JY: conceptualization, formal analysis, and writing – original. DK: methodology and formal analysis. SL, SHL and BA: Resources and data curation. TK: conceptualization, supervision, writing – review & editing. SP: conceptualization, supervision, writing – review & editing, and funding acquisition. All authors contributed to the article and approved the submitted version.

## Supplementary material

Supplementary Figure 1 Microbial diversity and composition in adjacent and tumor tissues of patients in the discovery set. **(A)** Alpha diversity. **(B)** Beta diversity. **(C)** Taxonomic composition at the phylum level. **(D)** Taxonomic composition at the genus level. **(E)** Taxonomic composition at the species level.

Supplementary Figure 2 Taxonomic composition of microbiomes. **(A–C)** Taxonomic composition at the phylum, genus, and species levels in the discovery set. **(D–F)** Taxonomic composition at the phylum, genus, and species levels in the validation set.

Supplementary Figure 3 Microbial diversity and abundance in adjacent and tumor tissues according to the presence of recurrence in male and female patients in the discovery set. **(A)** Alpha diversity. **(B)** Phylum level. **(C)** Genus level.

Supplementary Figure 4 Microbial diversity and abundance in tumor tissues according to the presence of recurrence in male and female patients in the validation set. **(A)** Alpha diversity. **(B)** Phylum level. **(C)** Genus level.
