# Supplementary material for: Tumor microbiome analysis provides prognostic value for patients with stage III colorectal cancer
Source: Front Oncol. 2023 Oct 26;13:1212812. doi: 10.3389/fonc.2023.1212812 (PMC10641399; doi:10.3389/fonc.2023.1212812)

Supplementary Figure 1

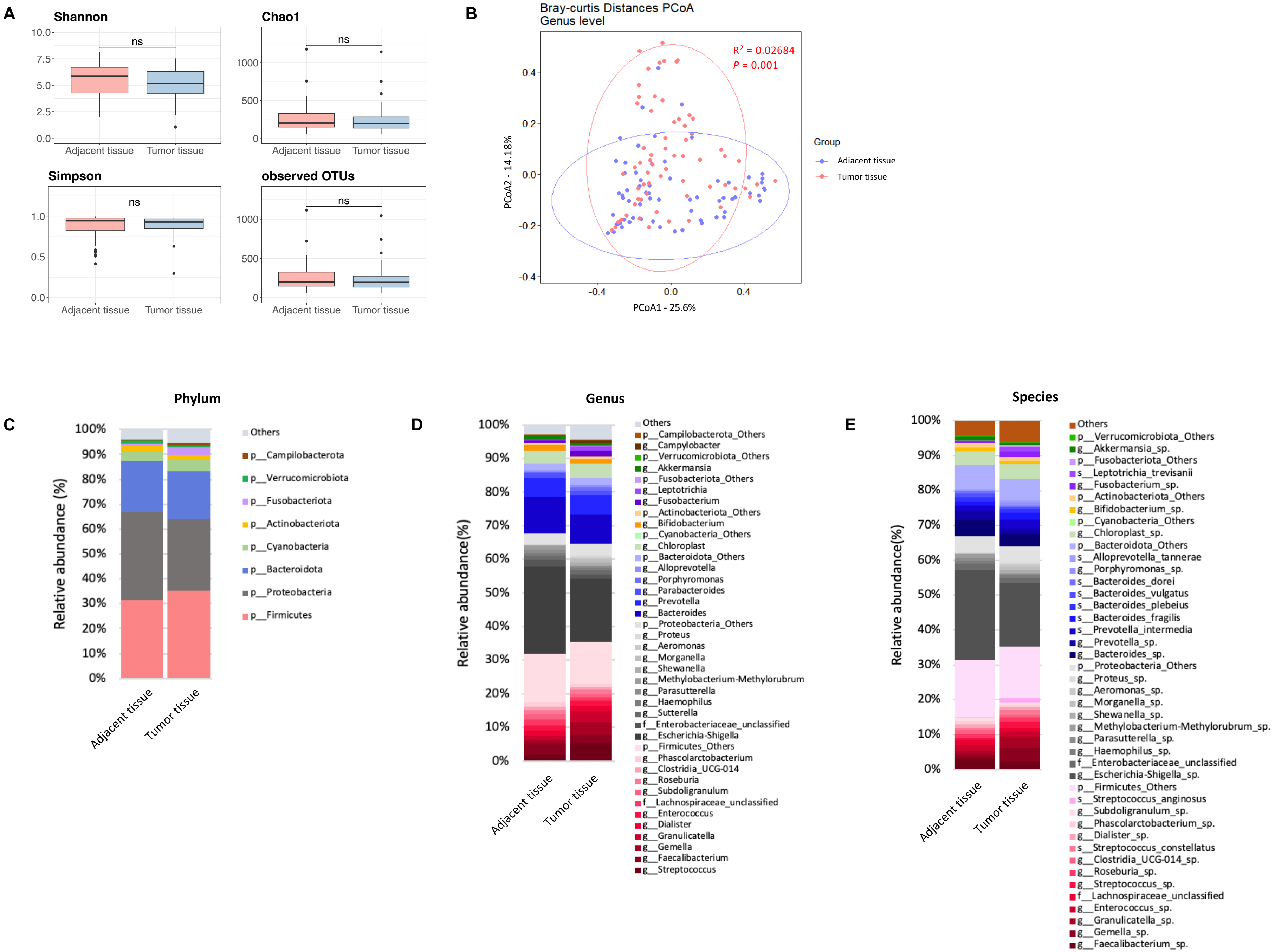

Supplementary Figure 2

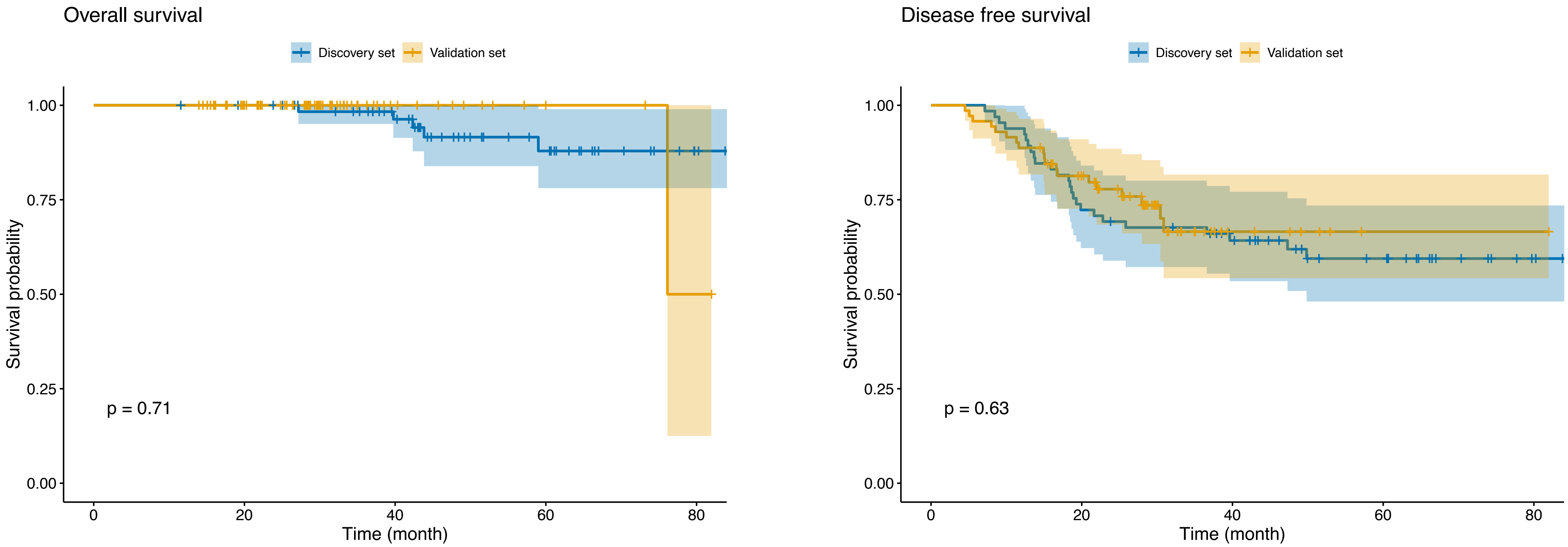

Supplementary Figure 3

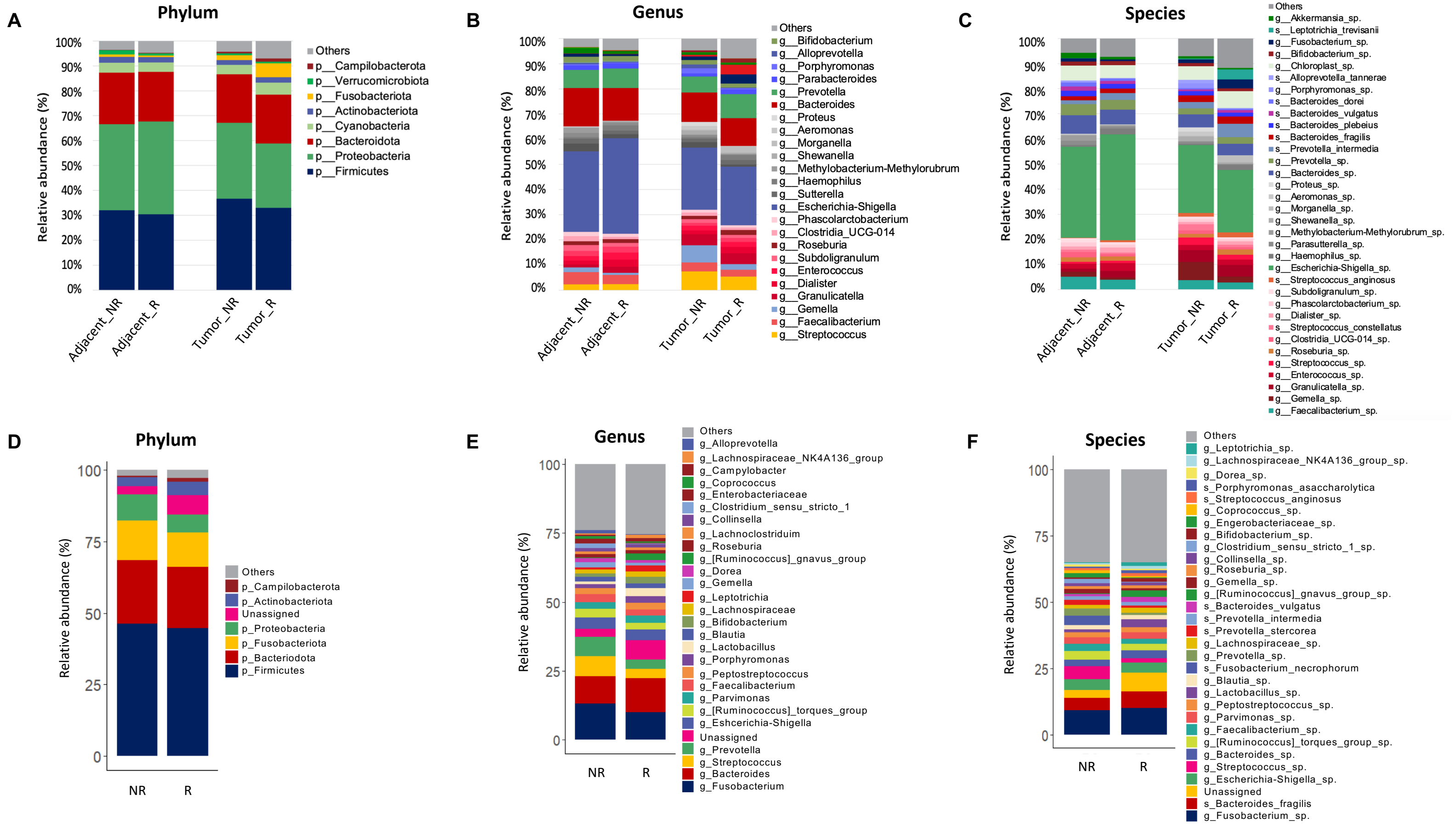

Supplementary Figure 4

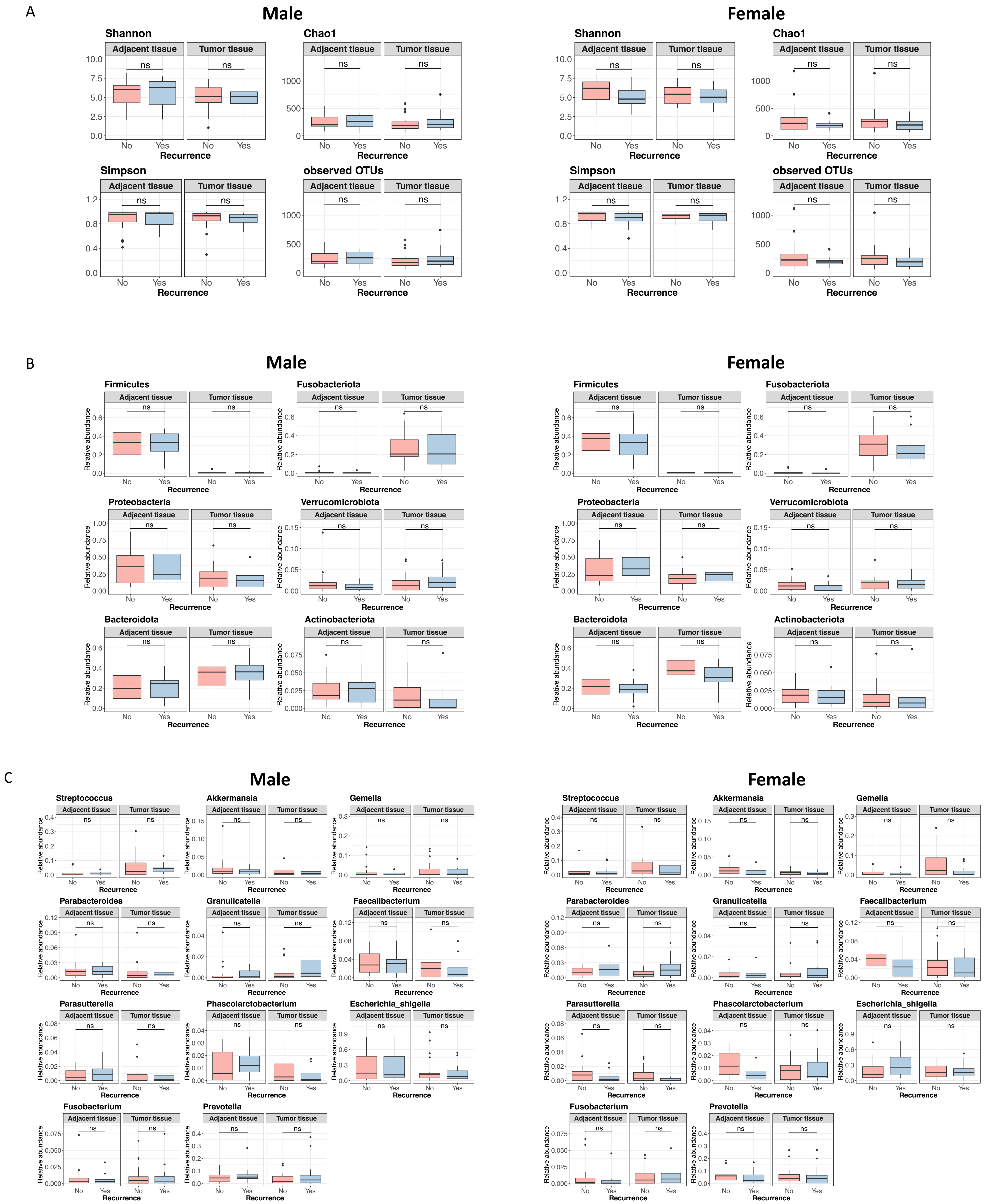

Supplementary Figure 5

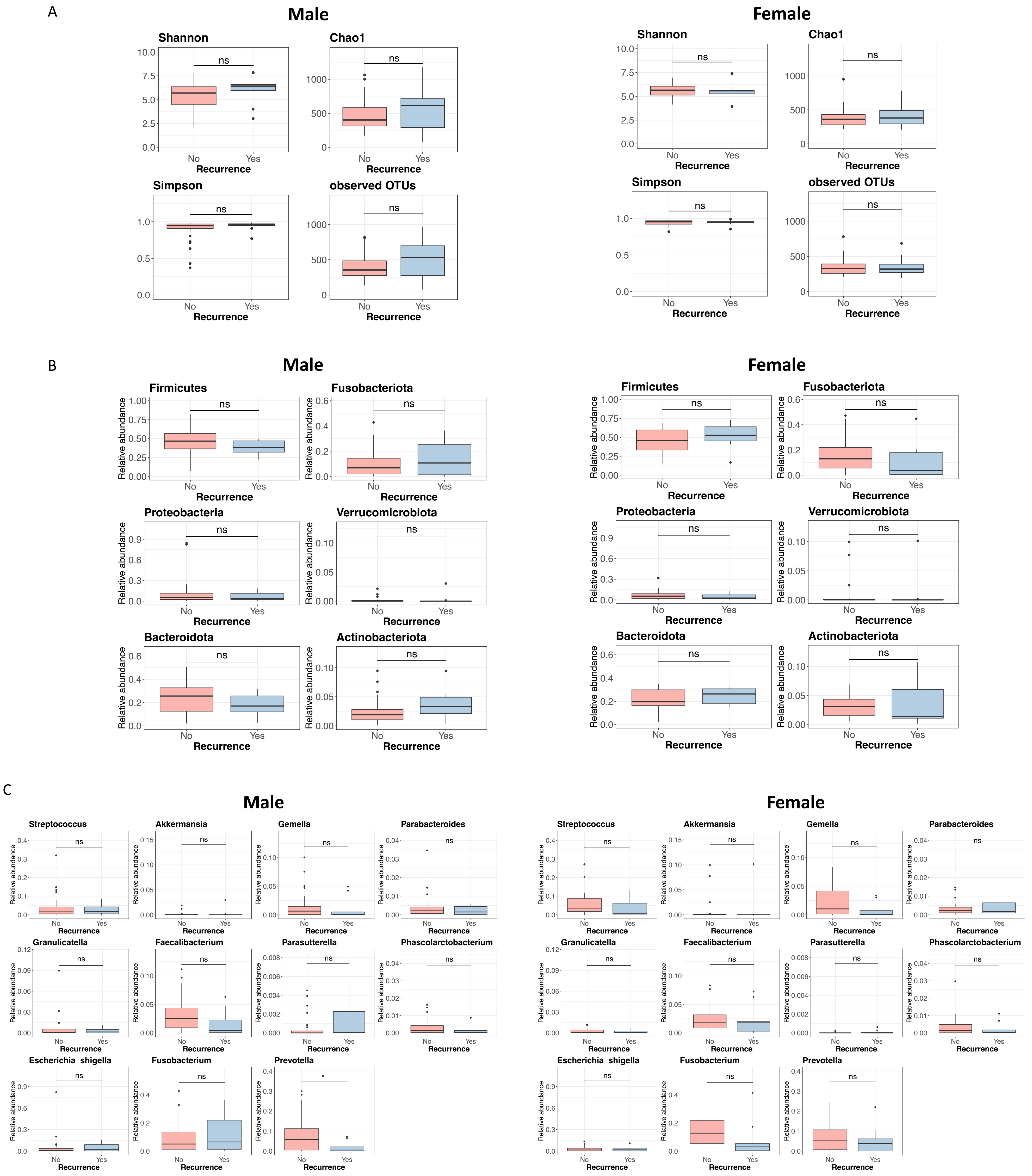

Supplement: Supplementary Figure 1 — Microbial diversity and composition in adjacent and tumor tissues of patients in the discovery set. (A) Alpha diversity. (B) Beta diversity. (C) Taxonomic composition at the phylum level. (D) Taxonomic composition at the genus level. (E) Taxonomic composition at the species level. [file Image_1.pdf]
