# Supplementary material for: Tumor microbiome analysis provides prognostic value for patients with stage III colorectal cancer
Source: Front Oncol. 2023 Oct 26;13:1212812. doi: 10.3389/fonc.2023.1212812 (PMC10641399; doi:10.3389/fonc.2023.1212812)
Supplement: Supplementary file 2 [file DataSheet_1.pdf]

Logistic regression analysis making the prediction model

JaeHyunKim

2023-03-03

Logistic regression analysis

```
# package
library(tidyverse)
library(lubridate)
library(fmsb)
library(carData)
library(car)
library(performance)
library(moonBook)
# data
data1<-read_csv("genus_select12.csv")
# date set-up
data1$dx_date<-ymd(data1$dx_date)
data1$dob<-ymd(data1$dob)
data1$dx_date<-as.Date(data1$dx_date)
data1$dob<-as.Date(data1$dob)
# conversion of numeric/factor
data1$recur<-as.numeric(data1$recur)
data1$recur<-as.factor(data1$recur)
data1$age<-(data1$dx_date-data1$dob)/365
data1$age<-as.numeric(data1$age)
data1$sex<-as.factor(data1$sex)
data1$tissue<-as.factor(data1$tissue)
data1$lympovas_inv<-as.numeric(data1$lympovas_inv)
data1$peri_inv<-as.numeric(data1$peri_inv)
data1$diff<-as.factor(data1$diff)
data1$stage_t<-as.numeric(data1$stage_t)
data1$stage_n<-as.numeric(data1$stage_n)
data1$locat<-as.factor(data1$locat)
data1$recur<-factor(data1$recur, labels=c('No','Yes'))

# data selection & rename
data2 <- data1 %>%
  filter(tissue=="1")
data3 <- data2 %>%
  rename(Recurrence=recur, Histology=diff, Age=age, CEA=cea,
         Lymphovascular_invasion=lympovas_inv, Perineural_invasion=peri_inv,
         Stage_T=stage_t, Stage_N=stage_n)

# Logistic regression analysis
```

```r
fit.multi <- glm(Recurrence~
                   Age+CEA+Histology+Lymphovascular_invasion+
                   Perineural_invasion+Stage_T+Stage_N+
                   Streptococcus+Akkermansia+
                   Gemella+Parabacteroides+Granulicatella+Faecalibacterium+
                   Parasutterella+Phascolarctobacterium+Escherichia_shigella+
                   Fusobacterium+Prevotella
                   , family=binomial, data=data3)
fit.final <- step(fit.multi, type="backward")
```

```
Start:  AIC=90.63
Recurrence ~ Age + CEA + Histology + Lymphovascular_invasion +
    Perineural_invasion + Stage_T + Stage_N + Streptococcus +
    Akkermansia + Gemella + Parabacteroides + Granulicatella +
    Faecalibacterium + Parasutterella + Phascolarctobacterium +
    Escherichia_shigella + Fusobacterium + Prevotella

                          Df Deviance    AIC
- Lymphovascular_invasion  1   52.715 88.715
- Histology                1   52.716 88.716
- Granulicatella           1   52.815 88.815
- Faecalibacterium         1   52.858 88.858
- Streptococcus            1   52.915 88.915
- Age                      1   53.042 89.042
- Fusobacterium            1   53.103 89.103
- Escherichia_shigella     1   53.146 89.146
- Stage_N                  1   53.151 89.151
- Akkermansia              1   53.431 89.431
- Prevotella               1   53.819 89.819
- Phascolarctobacterium    1   54.066 90.066
- Stage_T                  1   54.250 90.250
<none>                         52.633 90.633
- Parabacteroides          1   54.726 90.726
- Perineural_invasion      1   57.126 93.126
- CEA                      1   60.801 96.801
- Parasutterella           1   61.231 97.231
- Gemella                  1   62.769 98.769

Step:  AIC=88.71
Recurrence ~ Age + CEA + Histology + Perineural_invasion + Stage_T +
    Stage_N + Streptococcus + Akkermansia + Gemella + Parabacteroides +
    Granulicatella + Faecalibacterium + Parasutterella + Phascolarctobacterium +
    Escherichia_shigella + Fusobacterium + Prevotella

                        Df Deviance    AIC
- Histology              1   52.819 86.819
- Granulicatella         1   52.880 86.880
- Faecalibacterium       1   52.900 86.900
- Streptococcus          1   53.074 87.074
- Age                    1   53.197 87.197
- Fusobacterium          1   53.236 87.236
- Stage_N                1   53.245 87.245
- Escherichia_shigella   1   53.354 87.354
```

```
- Akkermansia           1   53.726 87.726
- Prevotella            1   53.862 87.862
- Phascolarctobacterium  1   54.085 88.085
- Stage_T               1   54.311 88.311
<none>                      52.715 88.715
- Parabacteroides       1   54.950 88.950
- Perineural_invasion   1   57.265 91.265
- CEA                   1   60.939 94.939
- Parasutterella        1   61.787 95.787
- Gemella               1   62.769 96.769

Step:  AIC=86.82
Recurrence ~ Age + CEA + Perineural_invasion + Stage_T + Stage_N +
    Streptococcus + Akkermansia + Gemella + Parabacteroides +
    Granulicatella + Faecalibacterium + Parasutterella + Phascolarctobacterium +
    Escherichia_shigella + Fusobacterium + Prevotella

                        Df Deviance    AIC
- Faecalibacterium       1   52.966 84.966
- Granulicatella         1   52.977 84.977
- Streptococcus          1   53.098 85.098
- Stage_N                1   53.248 85.248
- Fusobacterium          1   53.316 85.316
- Age                    1   53.353 85.353
- Escherichia_shigella   1   53.367 85.367
- Akkermansia            1   53.838 85.838
- Prevotella             1   53.998 85.998
- Phascolarctobacterium  1   54.126 86.126
- Stage_T                1   54.675 86.675
<none>                       52.819 86.819
- Parabacteroides        1   54.993 86.993
- Perineural_invasion    1   57.266 89.266
- CEA                    1   61.081 93.081
- Parasutterella         1   62.297 94.297
- Gemella                1   62.894 94.894

Step:  AIC=84.97
Recurrence ~ Age + CEA + Perineural_invasion + Stage_T + Stage_N +
    Streptococcus + Akkermansia + Gemella + Parabacteroides +
    Granulicatella + Parasutterella + Phascolarctobacterium +
    Escherichia_shigella + Fusobacterium + Prevotella

                        Df Deviance    AIC
- Granulicatella         1   53.166 83.166
- Streptococcus          1   53.236 83.236
- Stage_N                1   53.334 83.334
- Escherichia_shigella   1   53.420 83.420
- Fusobacterium          1   53.507 83.507
- Age                    1   53.577 83.577
- Phascolarctobacterium  1   54.153 84.153
- Akkermansia            1   54.224 84.224
- Prevotella             1   54.338 84.338
- Stage_T                1   54.675 84.675
<none>                       52.966 84.966
```

```
- Parabacteroides        1   55.233 85.233
- Perineural_invasion    1   57.956 87.956
- CEA                    1   61.335 91.335
- Gemella                1   63.272 93.272
- Parasutterella         1   63.796 93.796

Step:  AIC=83.17
Recurrence ~ Age + CEA + Perineural_invasion + Stage_T + Stage_N +
    Streptococcus + Akkermansia + Gemella + Parabacteroides +
    Parasutterella + Phascolarctobacterium + Escherichia_shigella +
    Fusobacterium + Prevotella

                        Df Deviance    AIC
- Streptococcus          1   53.266 81.266
- Stage_N                1   53.553 81.553
- Escherichia_shigella   1   53.638 81.638
- Age                    1   53.757 81.757
- Phascolarctobacterium  1   54.169 82.169
- Fusobacterium          1   54.317 82.317
- Akkermansia            1   54.372 82.372
- Prevotella             1   54.781 82.781
<none>                       53.166 83.166
- Stage_T                1   55.169 83.169
- Parabacteroides        1   55.481 83.481
- Perineural_invasion    1   57.996 85.996
- CEA                    1   61.789 89.789
- Gemella                1   63.351 91.351
- Parasutterella         1   63.883 91.883

Step:  AIC=81.27
Recurrence ~ Age + CEA + Perineural_invasion + Stage_T + Stage_N +
    Akkermansia + Gemella + Parabacteroides + Parasutterella +
    Phascolarctobacterium + Escherichia_shigella + Fusobacterium +
    Prevotella

                        Df Deviance    AIC
- Stage_N                1   53.595 79.595
- Escherichia_shigella   1   53.647 79.647
- Age                    1   53.894 79.894
- Phascolarctobacterium  1   54.289 80.289
- Fusobacterium          1   54.382 80.382
- Akkermansia            1   54.401 80.401
- Prevotella             1   55.159 81.159
<none>                       53.266 81.266
- Stage_T                1   55.584 81.584
- Parabacteroides        1   55.919 81.919
- Perineural_invasion    1   58.310 84.310
- CEA                    1   61.838 87.838
- Parasutterella         1   63.888 89.888
- Gemella                1   65.828 91.828

Step:  AIC=79.59
Recurrence ~ Age + CEA + Perineural_invasion + Stage_T + Akkermansia +
    Gemella + Parabacteroides + Parasutterella + Phascolarctobacterium +
```

```
    Escherichia_shigella + Fusobacterium + Prevotella

                        Df Deviance    AIC
- Escherichia_shigella   1   54.039 78.039
- Age                    1   54.235 78.235
- Fusobacterium          1   54.522 78.522
- Phascolarctobacterium  1   54.607 78.607
- Prevotella             1   55.206 79.206
- Akkermansia            1   55.420 79.420
<none>                       53.595 79.595
- Stage_T                1   55.869 79.869
- Parabacteroides        1   56.435 80.435
- Perineural_invasion    1   58.899 82.899
- CEA                    1   62.031 86.031
- Parasutterella         1   64.076 88.076
- Gemella                1   68.069 92.069

Step:  AIC=78.04
Recurrence ~ Age + CEA + Perineural_invasion + Stage_T + Akkermansia +
    Gemella + Parabacteroides + Parasutterella + Phascolarctobacterium +
    Fusobacterium + Prevotella

                        Df Deviance    AIC
- Age                    1   54.923 76.923
- Fusobacterium          1   55.299 77.299
- Phascolarctobacterium  1   55.352 77.352
- Akkermansia            1   55.632 77.632
<none>                       54.039 78.039
- Prevotella             1   56.280 78.280
- Stage_T                1   56.419 78.419
- Parabacteroides        1   56.723 78.723
- Perineural_invasion    1   59.731 81.731
- CEA                    1   62.107 84.107
- Parasutterella         1   64.311 86.311
- Gemella                1   68.145 90.145

Step:  AIC=76.92
Recurrence ~ CEA + Perineural_invasion + Stage_T + Akkermansia +
    Gemella + Parabacteroides + Parasutterella + Phascolarctobacterium +
    Fusobacterium + Prevotella

                        Df Deviance    AIC
- Phascolarctobacterium  1   55.806 75.806
- Fusobacterium          1   56.150 76.150
- Akkermansia            1   56.829 76.829
<none>                       54.923 76.923
- Prevotella             1   57.009 77.009
- Parabacteroides        1   57.779 77.779
- Stage_T                1   58.172 78.172
- Perineural_invasion    1   59.965 79.965
- CEA                    1   62.399 82.399
- Parasutterella         1   64.316 84.316
- Gemella                1   68.166 88.166
```

```
Step:  AIC=75.81
Recurrence ~ CEA + Perineural_invasion + Stage_T + Akkermansia +
    Gemella + Parabacteroides + Parasutterella + Fusobacterium +
    Prevotella

                      Df Deviance    AIC
- Fusobacterium        1   56.980 74.980
- Akkermansia          1   57.212 75.212
<none>                     55.806 75.806
- Prevotella           1   58.283 76.283
- Stage_T              1   58.789 76.789
- Parabacteroides      1   59.041 77.041
- Perineural_invasion  1   60.450 78.450
- CEA                  1   62.403 80.403
- Parasutterella       1   64.947 82.947
- Gemella              1   68.761 86.761

Step:  AIC=74.98
Recurrence ~ CEA + Perineural_invasion + Stage_T + Akkermansia +
    Gemella + Parabacteroides + Parasutterella + Prevotella

                      Df Deviance    AIC
- Akkermansia          1   58.899 74.899
<none>                     56.980 74.980
- Prevotella           1   59.535 75.535
- Parabacteroides      1   59.729 75.729
- Stage_T              1   59.821 75.821
- Perineural_invasion  1   61.684 77.684
- CEA                  1   63.443 79.443
- Parasutterella       1   66.651 82.651
- Gemella              1   70.141 86.141

Step:  AIC=74.9
Recurrence ~ CEA + Perineural_invasion + Stage_T + Gemella +
    Parabacteroides + Parasutterella + Prevotella

                      Df Deviance    AIC
<none>                     58.899 74.899
- Parabacteroides      1   61.100 75.100
- Stage_T              1   61.794 75.794
- Prevotella           1   62.375 76.375
- Perineural_invasion  1   62.468 76.468
- CEA                  1   65.097 79.097
- Parasutterella       1   68.953 82.953
- Gemella              1   71.279 85.279
```

```
fit.final
```

```
Call:  glm(formula = Recurrence ~ CEA + Perineural_invasion + Stage_T +
    Gemella + Parabacteroides + Parasutterella + Prevotella,
    family = binomial, data = data3)

Coefficients:
```

```
        (Intercept)                 CEA  Perineural_invasion
           -4.29796             0.04667              1.47743
            Stage_T             Gemella      Parabacteroides
            1.08028           -33.38073             28.07568
     Parasutterella          Prevotella
         -141.75533             7.85802

Degrees of Freedom: 64 Total (i.e. Null);  57 Residual
Null Deviance:      86.62
Residual Deviance: 58.9     AIC: 74.9
```

```
summary(fit.final)
```

```
Call:
glm(formula = Recurrence ~ CEA + Perineural_invasion + Stage_T +
    Gemella + Parabacteroides + Parasutterella + Prevotella,
    family = binomial, data = data3)

Deviance Residuals:
    Min       1Q   Median       3Q      Max
-1.8902  -0.7870  -0.2227   0.8438   1.7214

Coefficients:
                      Estimate Std. Error z value Pr(>|z|)
(Intercept)           -4.29796    2.18834  -1.964  0.04953 *
CEA                    0.04667    0.02348   1.988  0.04685 *
Perineural_invasion    1.47743    0.81917   1.804  0.07130 .
Stage_T                1.08028    0.66699   1.620  0.10531
Gemella              -33.38073   12.54451  -2.661  0.00779 **
Parabacteroides       28.07568   20.29487   1.383  0.16655
Parasutterella      -141.75533   62.67142  -2.262  0.02370 *
Prevotella             7.85802    4.71908   1.665  0.09588 .
---
Signif. codes:  0 '***' 0.001 '**' 0.01 '*' 0.05 '.' 0.1 ' ' 1

(Dispersion parameter for binomial family taken to be 1)

    Null deviance: 86.616  on 64  degrees of freedom
Residual deviance: 58.899  on 57  degrees of freedom
AIC: 74.899

Number of Fisher Scoring iterations: 6
```

```
extractOR(fit.final)
```

```
                              OR  lcl          ucl      p
(Intercept)         1.000000e-02 0.00 9.900000e-01 0.0495
CEA                 1.050000e+00 1.00 1.100000e+00 0.0468
Perineural_invasion 4.380000e+00 0.88 2.182000e+01 0.0713
Stage_T             2.950000e+00 0.80 1.089000e+01 0.1053
Gemella             0.000000e+00 0.00 0.000000e+00 0.0078
Parabacteroides     1.559951e+12 0.00 2.938587e+29 0.1665
```

```
Parasutterella      0.000000e+00 0.00 0.000000e+00 0.0237
Prevotella          2.586380e+03 0.25 2.688933e+07 0.0959
```

```r
NagelkerkeR2(fit.final)
```

```
$N
[1] 65

$R2
[1] 0.4715475
```

```r
vif(fit.final)
```

```
               CEA Perineural_invasion             Stage_T             Gemella 
          1.206262            1.212216            1.089850            1.495838 
    Parabacteroides      Parasutterella          Prevotella 
          1.147815            1.189068            1.131573 
```

```r
AIC(fit.final)
```

```
[1] 74.89945
```
